# Polar vortices on Earth and Mars: A comparative study of the climatology and variability from reanalyses

**DOI:** 10.1002/qj.2376

**Published:** 2014-06-12

**Authors:** D M Mitchell, L Montabone, S Thomson, P L Read

**Affiliations:** a Atmospheric, Oceanic and Planetary Physics, University of OxfordUK; b Laboratoire de Météorologie Dynamique, Université Pierre et Marie CurieParis, France; c Space Science InstituteBoulder, CO, USA; d Department of Applied Mathematics and Theoretical Physics, University of CambridgeUK

**Keywords:** Mars, Martian atmosphere, polar vortex, TEM diagnostics

## Abstract

Polar vortices on Mars provide case-studies to aid understanding of geophysical vortex dynamics and may help to resolve long-standing issues regarding polar vortices on Earth. Due to the recent development of the first publicly available Martian reanalysis dataset (MACDA), for the first time we are able to characterise thoroughly the structure and evolution of the Martian polar vortices, and hence perform a systematic comparison with the polar vortices on Earth. The winter atmospheric circulations of the two planets are compared, with a specific focus on the structure and evolution of the polar vortices. The Martian residual meridional overturning circulation is found to be very similar to the stratospheric residual circulation on Earth during winter. While on Earth this residual circulation is very different from the Eulerian circulation, on Mars it is found to be very similar. Unlike on Earth, it is found that the Martian polar vortices are annular, and that the Northern Hemisphere vortex is far stronger than its southern counterpart. While winter hemisphere differences in vortex strength are also reported on Earth, the contrast is not as large. Distinctions between the two planets are also apparent in terms of the climatological vertical structure of the vortices, in that the Martian polar vortices are observed to decrease in size at higher altitudes, whereas on Earth the opposite is observed. Finally, it is found that the Martian vortices are less variable through the winter than on Earth, especially in terms of the vortex geometry. During one particular major regional dust storm on Mars (Martian year 26), an equatorward displacement of the vortex is observed, sharing some qualitative characteristics of sudden stratospheric warmings on Earth.

## 1. Introduction

Polar vortices are hemispheric-scale rotations of the polar air mass located approximately over a planet's poles. In the Earth's stratosphere, the strong latitudinal temperature gradients observed during polar winter are associated with circumpolar westerly winds at ∼60°N and ∼60°S. In summer the stratospheric temperature gradient is reversed (such that polar temperatures are higher than equatorial temperatures) and weaker, and so no well-defined polar vortex is formed. Distinct polar vortices have also been observed on other planetary bodies, including Venus, Mars, Jupiter, Saturn and Titan, (e.g. Wilson, 1997; Teanby *et al.*, 2008; Barbosa Aguiar *et al.*, 2010; Luz *et al.*, 2011).

Over the recent past the importance of the terrestrial stratospheric polar vortices has become ever more apparent. For instance, the strong westerly winds on the edge of the vortex can act as a barrier to meridional transport of chemical species. This has contributed to spring and early wintertime ozone depletion in the SH (all acronyms are given in Table A1) and to a lesser extent in the NH. One particularly important aspect of polar vortex behaviour is that, during the winter, the polar vortex of westerly winds can break down dynamically through interactions with Rossby waves (Andrews *et al.*, 1987). These events are known as SSWs because they are accompanied by a local rise of stratospheric temperature by tens of degrees. They have also been shown to be important in understanding surface circulation patterns and weather (e.g. Baldwin and Dunkerton, 2001; Mitchell *et al.*, 2013). SSWs are of particular importance when considering how climate in the mid–high northern latitudes varies on seasonal time-scales (e.g. Sigmond *et al.*, 2013) and on decadal to multi-decadal time-scales (e.g. Scaife *et al.*, 2012).

Since the first observed SSW in 1952 (Scherhag, 1952), atmospheric scientists have sought to understand whether and how these events might influence conditions at the surface. Many theories have been put forward, which include planetary wave reflection and refraction at a critical line (Perlwitz and Graf, 1995; Perlwitz and Harnik, 2003), PV-induced perturbations to the tropopause and the subsequent tropospheric PV stretching (Ambaum and Hoskins, 2002; Mitchell *et al.*, 2013) and changes in upper-level baroclinicity and transient eddies (Wittman *et al.*, 2007; Simpson *et al.*, 2009; Scaife *et al.*, 2012). In reality it is more likely to be the result of a mixture of these mechanisms, but even so no clear consensus has emerged.

Mars also exhibits polar warming in its upper atmosphere (40–70 km altitude) (Barnes and Hollingsworth, 1987; Wilson, 1997). However, we will distinguish events of rapid polar warming*, and what we shall term *seasonal polar warming*. The seasonal polar warming in the NH tends to be more pronounced than in the SH because in the NH autumn and winter, when Mars is closer to perihelion, dust (a strong absorber of short-wave radiation on Mars) is lifted by larger near-surface winds, and becomes the main driver of the circulation (Newman *et al.*, 2002a, 2002b). In some MYs, the polar warming also exhibits a transient component in the NH, linked to the evolution of major dust storm events at sensitive times and locations. The dust is lifted due to various dynamical phenomena, normally associated with baroclinic waves or western boundary currents. This has the effect of enhancing the seasonal component substantially because dust in the summer hemisphere absorbs short-wave radiation, and so increases the Equator–Pole temperature gradient in the winter hemisphere. During such times the vortex region can warm by tens of degrees over a few days (hereafter an RPW event). Such events have been observed as far back as 1971 by Mariner 9 (Deming *et al.*, 1986; Jakosky and Martin, 1987). The anomalous structure and evolution of these Martian RPW events have been hard to characterise because of inhomogeneities in space-based observations, and the authors know of no comprehensive observational studies. However, modelling studies have attempted to reproduce Martian polar warmings (Barnes and Hollingsworth, 1987; Wilson, 1997). Most notably, Barnes and Hollingsworth (1987) used a simplified nonlinear model, previously used to simulate SSW events on Earth, to successfully simulate an SSW event on Mars. They argued that planetary-scale Rossby waves could be responsible for the warmings observed on Mars, in much the same way as on Earth. However, they also demonstrated that such warming events must involve only zonal wavenumber-1 scale waves, as opposed to Earth's SSW events, which can involve wavenumbers 1 and 2.

Wilson (1997) proposed an alternative mechanism which linked a model-generated RPW event to global-scale dust storm activity, which was emulated by injecting dust uniformly in the lowermost model level. They showed that the evolving aerosol distribution could alter the symmetry of the Martian Hadley circulation about the Equator. This event, in combination with thermal tides, allows for a more poleward extent of the circulation and more downwelling over the Pole. Forget confirmed the important role of the Martian Hadley circulation, using a then state-of-the-art MGCM. They were able to demonstrate that thermal inversions were present at around 60–70 km over the winter Pole, and to confirm that adiabatic heating caused by downwelling motions over the polar regions is one of the main drivers of the polar warming.

More recently, Kuroda *et al.* (2009) used a global climate model to investigate the Martian meridional circulation during ‘high dust storm’ and ‘low dust storm’ conditions. They showed that the enhanced poleward extent of the overturning circulation was not solely due to tides, but equally due to planetary waves, resolved gravity waves and geostrophic eddies. In particular, they showed that the mean atmospheric state during high dust storm conditions allowed for more generation, and more favourable vertical propagation, of zonal wavenumber-1 planetary waves, the primary driver of SSW events on Earth.

Other than those discussed here, there have been only a few indirect studies on Martian polar vortices and associated RPW events. With the advent of Martian reanalyses it is therefore timely to examine the climatological nature of the Martian polar vortices and compare them with their terrestrial counterparts. In so doing, we hope to further our understanding of geophysical vortex dynamics by comparison with a planet which shows similarities to Earth in the characteristics of its atmospheric circulation. It would also be desirable to determine the limits within which Mars can be used as an analogy to the Earth for inferring properties of terrestrial vortex dynamics. For example, on Earth, only one known SSW has been observed in the SH. This event sparked a wealth of research comparing with the known NH SSW events (Varotsos, 2002; Krüger *et al.*, 2005; Newman and Nash, 2005), with theories suggesting dominant roles for the interaction of eastward-travelling waves with quasi-stationary planetary waves forced in the troposphere (Krüger *et al.*, 2005) as well as enhanced tropospheric cyclogenesis (A. O'Neill, 2013; personal communication). Importantly, when such unprecedented events occur, great advances in a field can be made. Due to the possible presence of SSW-like events on Mars, in this study we test whether Mars is a suitable analogue to Earth by considering both the mean state and variability of the vortices. Specific RPW case-studies will be addressed in future work.

The article is organised as follows; section 2 gives an overview of the reanalysis datasets used for each planet and defines the various analysis diagnostics used. The analysis is performed in section 3, which first compares the atmospheric mean state, then the polar vortices. Finally, conclusions are given in section 4. The Appendix includes some common terms.

## 2. Description of reanalyses

Retrospective analyses (or *reanalyses*) combine state-of-the-art, high-resolution models with incomplete and noisy observations to produce a uniform-in-time, regularly sampled estimate of the atmospheric state. On Earth there are many such reanalyses to choose from. Here, we use the MERRA as it has high vertical resolution (72 vertical levels), a high model lid height (0.01 hPa) and spans a period of more than 30 years from 1979 to 2011 (Rienecker *et al.*, 2011). Data are available at 6 h intervals on a 1/2° latitude × 2/3° longitude grid. We also used the ECMWF ERA-Interim reanalysis (Dee *et al.*, 2011) to confirm our results. The two reanalyses agree exceptionally well for all the diagnostics used here, so we display results only using MERRA.

For Mars, only one reanalysis dataset is publicly available to date, although other reanalysis projects are ongoing (e.g. Greybush *et al.*, 2012). We use the MACDA reanalysis for MGS/TES observations, spanning the period 1999–2004 (which corresponds to the period from late northern summer in MY 24 to late northern spring in MY 27)†. This dataset, publicly available online via the British Atmospheric Data Centre (Montabone *et al.*, 2011), is described in Montabone. The MACDA reanalysis uses the Analysis Correction scheme (Lorenc *et al.*, 1991) in a MGCM based on the UK spectral version of an earlier version of the Laboratoire de Météorologie Dynamique MGCM (Forget *et al.*, 1999). The assimilated observations consist of retrieved thermal profiles, and column dust optical depths from MGS/TES infrared atmospheric soundings in nadir view (Smith, 2004). The Mars atmospheric reanalysis has 25 vertical levels in terrain-following (*σ*) coordinates, approximately from 5 m up to 100 km altitude, corresponding to average pressures between ∼6.1 hPa and ∼3.4×10^−4^ hPa (the three uppermost levels are used as a sponge layer), and data are output at 2 h intervals on a 5° latitude × 5° longitude grid. As this is a relatively coarse resolution, we would not expected the reanalysis to reproduce fine-scale structure associated with, for example, filamentation on the vortex edge. Therefore we do not consider such features in this study.

### 2.1. Mean state diagnostics

To compare the mean state of the Martian and terrestrial atmospheres, we first examine zonal mean temperature and wind. We also consider the Eulerian meridional stream function, *ψ*, defined as


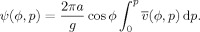
(1)

Here *ψ*(*ϕ,p*) is the mean meridional mass stream function as seen at pressure *p* and latitude *ϕ*. Additionally, *a* is the planetary radius, *g* is the acceleration due to gravity, and 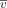 is the zonal mean meridional wind. Positive values of *ψ*(*ϕ,p*) indicate clockwise circulation when plotted with south on the left and north on the right. The quantity *ψ*(*ϕ,p*) is calculated using the trapezium rule for the integration, assuming *v*(*p* = 0) = 0.

### 2.2. Vortex-centric diagnostics

A common issue in comparative planetology is the choice of diagnostics to give meaningful comparisons. Distance from the Sun, size of the planet and orbital ellipticity are some of the many parameters that can make planetary atmospheres non-trivial to compare. Here, we use vortex-centric diagnostics to allow the relative characteristics of the vortices to be compared. To study the polar vortices we use Ertel's PV, *q*, which is defined as


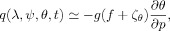
(2)

where *ζ*_*θ*_ is the vertical component of relative vorticity evaluated on a *θ* surface, where *θ* is potential temperature. As the magnitude of PV varies dramatically with *p*, this can often make comparisons of PV maps at different altitudes problematic. To circumvent this issue, Lait (1994) devised the scaling


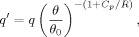
(3)

where *θ*_0_ is an arbitrarily chosen potential temperature reference level (we use 200 K for both planets throughout the analysis), *C*_*p*_ is specific heat at constant pressure and *R* is the universal gas constant. Note that the ratio *C*_*p*_*/R* is assumed constant and takes the value 4.4 on Mars, and 3.5 on Earth. Note also that this scaling is valid, strictly speaking, for an isothermal atmosphere. For a non-isothermal atmosphere, the scaling might affect the apparent strength of the PV field with altitude. Nevertheless, our main purpose is to compare the morphology of the polar vortices on Earth and Mars, rather than the vertical strength of the PV field, therefore the Lait scaling satisfies our requirement.

The polar vortices are clearly observable as a high positive (or negative in the SH) PV region in the polar winter. As PV is conserved on isentropic surfaces under frictionless and adiabatic flow, Waugh (1997) showed that an ellipse representing the shape of the vortex at a specific height could be tracked throughout winter. Harvey *et al.* (2009) further showed that PV at much higher altitudes (∼3000 K, ∼60 km) was not appropriate for this type of analysis. As our analyses are confined to altitudes lower than this, it is valid to use the diagnostics described in Waugh (1997) to measure key geometric characteristics of the polar vortex, such as the vortex area, ellipticity and centroid location. First, we define the vortex edge as the value of PV which represents the sharpest PV gradient in an equivalent latitude frame, 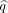. Note that, if there are two maxima, we choose the maximum which coincides with the strongest winds (as in Nash *et al.*, 1996). We then define the vortex area, *A*, as the area enclosed by the contour representing the vortex edge. By defining the polar vortex as the region where 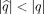 (note the absolute value is used since PV is negative in the SH), we can define the elliptical diagnostics equations (Waugh 1997). This is most conveniently performed in a Cartesian frame and we use a Lambert projection‡ to transform. The elliptical diagnostics are based on 2D moments of the polar vortex PV distribution (i.e. where 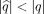). The moments of order *m* in the *x*-direction and *n* in the *y*-direction are given as


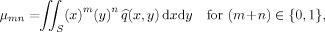
(4)


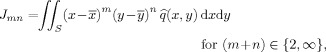
(5)

where


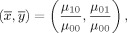
(6)

where *S* is the surface encompassed by 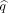. In some circumstances, there will be more than one region encompassed by 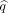, i.e. when the terrestrial vortex splits into two child vortices centred over Canada and Siberia. No such event happens on Mars over the period studied. During vortex-splitting events, we consider only the largest vortex (always the vortex centred over Siberia), and apply the diagnostics using the method detailed above. Note that other studies have considered the child vortices individually (e.g. Matthewman *et al.*, 2009; Mitchell *et al.*, 2011, 2013), however their focus was specifically on these types of events, rather than on the climatology as a whole.

From Eq. (5) the ellipse orientation (defined as the angle between the ellipse major axis and the prime meridian in the clockwise direction), *χ*, and aspect ratio, *r*, are defined following the exact methodology of Matthewman *et al.* (2009):


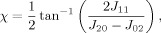
(7)


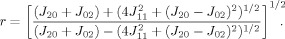
(8)

## 3. Comparative climatology of polar vortices

### 3.1. The winter atmosphere

In the absence of dynamical influences, one would expect polar vortices to be strongest around the winter solstice, when the Equator-to-Pole temperature gradients are highest. However, there will be a slight time lag between solstice and the lowest temperatures, which will be longer on Earth than Mars because oceans serve as a reservoir of heat. Therefore, in this study we explicitly study the climatological winter hemispheres of the two planets. Read (2011) gives an annual mean analysis of the dynamics and circulation of the two planets. Table 1 compares some of the important parameters of the two planets.

**Table 1:** A comparison of orbital and atmospheric parameters for Earth and Mars, taken from http://nssdc.gsfc.nasa.gov/planetary/factsheet/marsfact.html. Radiative time-scales are given for the lower stratosphere to lower mesosphere on Earth, and for the surface to lower mesosphere on Mars (e.g. Goody and Belton, 1967)

	Earth	Mars
Sidereal year (days)	365.256	686.980
Length of planetary day (h)	24	24.66
Orbit eccentricity	0.0167	0.0935
Mean planetary radius (km)	6371.0	3389.5
Surface gravity	9.80	3.71
Main atmospheric constituent	N_2_ (78%)	CO_2_ (95%)
Mean surface pressure (hPa)	1013.0	6.1
Scale height (km)	7.5	10.8
C_*p*_*/R* ratio	3.5	4.5
Rossby deformation radius (km)	1100	920
Radiative time-scale	30–2 (days)	2–0.05 (sols)

**Figure 1:**
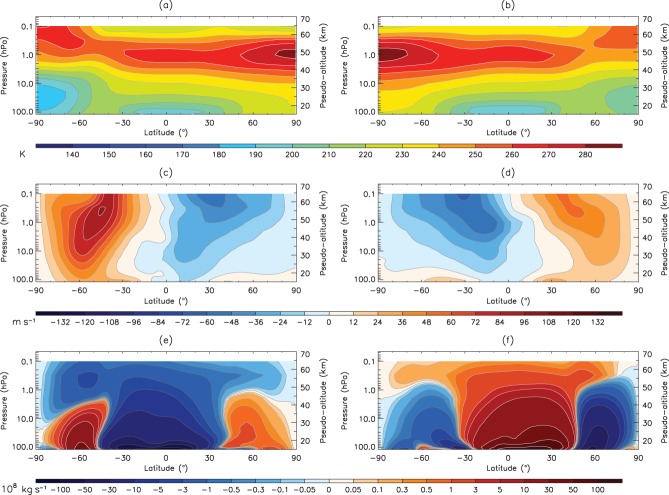
The climatological (a, b) zonal mean temperature, (c, d) zonal wind speed, and (e, f) meridional mass stream function for the Earth's stratosphere and lower mesosphere. Data are extracted from the MERRA reanalysis over the period 1979–2011. (a, c, e) are time averaged over June–July–August (JJA), and (b, d, f) over December–January–February (DJF). The pseudo-altitude scale has been calculated using the formula *z*_*p*_ = −*H* log(*p/p*_*ref*_), where *H* = 7 km (reference Earth scale-height) and *p*_*ref*_ ∼ 1013 hPa.

Figure 1 shows the zonal mean temperature, the zonal mean zonal wind and the meridional MSF for SH winter and NH winter on Earth (we exclude the troposphere as the stratosphere is found to be a better analogy to the Mars atmosphere). Notably, the zonal mean temperature increases with height (Figure 1(a, b)) from the tropopause (∼100 hPa) to the stratopause (∼1 hPa), and this is principally due to absorption of ultraviolet radiation by stratospheric ozone. More importantly for the formation of the polar vortices is the reversed latitudinal temperature gradient in the winter hemisphere compared to the summer hemisphere. Due to the small amount of solar radiation absorption at high latitudes in the winter hemisphere, a particularly strong meridional temperature gradient is present. Consequently, via thermal wind balance, a strong vertical shear in the zonal wind (Figure 1(c, d)) is observed in the winter hemisphere, which is associated with a strong westerly circumpolar stratospheric jet (the core of which roughly corresponds to the polar vortex edge, defined as the location of the maximum 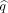 gradient). The jet tilts equatorward with height, primarily due to the influence of small-scale gravity waves on the circulation near the stratopause. The stratospheric jet in the SH (Figure 1(c)) is notably stronger than in the NH (Figure 1(d)), and this is due to a high influx of vertically propagating Rossby waves from the troposphere into the stratosphere at mid–high northern latitudes, which is greatly reduced in the SH (due to the absence of large orographic features). The interaction of these waves with the stratospheric mean state leads to a ubiquitous reduction of the winter zonal winds in the NH, and generally a less stable polar vortex than in the SH (Andrews *et al.*, 1987). The strength of the stratospheric jets can in part determine the extent to which atmospheric tracers (such as ozone) can reach high latitudes. The MSF (Eq. (1)) gives an Eulerian mean measure of latitudinal transport and here we use the convention that red contours indicate clockwise flow, and blue contours indicate anti-clockwise flow (Figure 1(e, f)). The meridional circulation is cross-equatorial, and strongest in the winter hemisphere and counter-rotating cells are observed in the polar regions. The primary cross-equatorial circulation also has a notably distinct lower branch (below ∼ 30 hPa) and upper branch. In some ways, the lower branch can be thought of as an extension of the residual circulation of the tropospheric Ferrell cell (Vallis, 2006).

**Figure 2:**
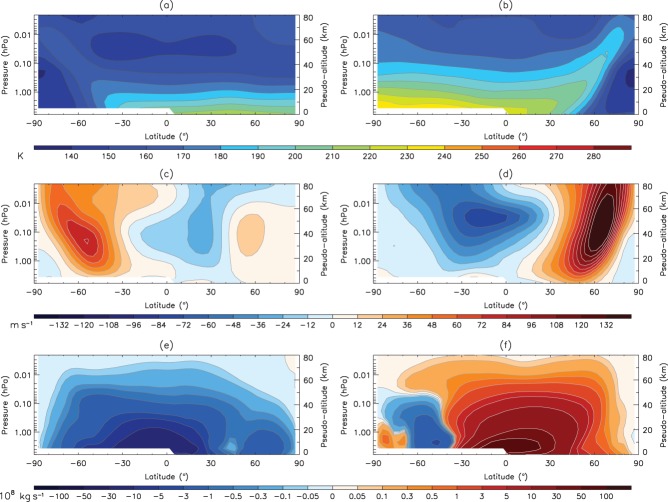
The climatological (a, b) zonal mean temperature, (c, d) zonal wind speed, and (e, f) meridional mass stream function for Mars' troposphere and lower mesosphere. Data are extracted from the MACDA reanalysis during the period 1999–2004 (MYs 24–26). (a, c, e) are time averaged around the northern summer solstice (solar longitude 45°–135°), and (b, d, f) are time averaged around the northern winter solstice (solar longitude 225°–315°). The pseudo-altitude scale has been calculated using the formula *z*_*p*_ = −*H* log(*p/p*_*ref*_), where *H* = 10 km (reference Martian scale height) and *p*_*ref*_ ∼ 6.1 hPa. Due to the elevated Martian topography in the SH, the lowest pressure levels might be located below the surface. We do not plot zonal mean values at a particular latitude and pressure level when more than half of the longitude points have missing values.

Of particular interest for this study is how the climatological winter atmospheric state of Earth compares with Mars, where winter on Mars is defined as *L*_*s*_ = 45°–135° for the SH, and *L*_*s*_ = 225°–315° for the NH. Figure 2 shows the same plot but for the Martian troposphere and lower mesosphere, which is where the polar vortices are observed (note that Mars does not have a stratospheric temperature inversion). As on Earth, the zonal mean temperature (Figure 2(a, b)) has a stronger Equator-to-Pole temperature gradient in the winter hemisphere than in the summer hemisphere. This gives rise to strong extratropical jets which are notably more intense in the NH winter (Figure 2(d)) than in the SH winter (Figure 2(c)). Unlike on Earth where the asymmetry between the stratospheric jet strengths in the two hemispheres arises primarily due to Rossby wave interactions with the stratospheric mean state, on Mars this asymmetry is likely to arise as a combined effect of the topographic asymmetry between the two hemispheres (which is greatly enhanced in the SH), the elliptical nature of Mars' orbit, and, by consequence, the seasonal and hemispheric asymmetries in the distribution of dust. Thus the mean strength of the jet maximum is around 70 m s^−1^ in the SH winter compared to over double this value in the NH, and this contrast between hemispheres is greater than observed on Earth (as well as being in the opposite hemispheres). The jet also appears to be more tilted towards the Pole with height than Earth (Figure 2(c, d)), and this is consistent across both hemispheres. While the fundamental reason for this is unclear, it is probably to do with differing amounts of momentum deposition from gravity waves in the jet region on Mars compared to Earth.

Finally, the winter averaged Eulerian MSF (Figure 2(e, f)) is clearly cross-equatorial on Mars, and shares many similar characteristics to the stratospheric Brewer–Dobson circulation (Brewer, 1949), most notably in the poleward extent of the circulation and the maximum in MSF strength associated with the winter solstice (consistent with Medvedev and Hartogh, 2007). However, the formation of the main meridional cell is primarily due to differential solar forcing between the Equator and Poles, much like that of the Hadley cell on Earth. Due to the short radiative time constant and absence of oceans (which, on Earth, help moderate seasonal temperature variations), the Martian MSF can vary dramatically throughout the annual cycle (Read and Lewis, 2004; Read, 2011).

**Figure 3:**
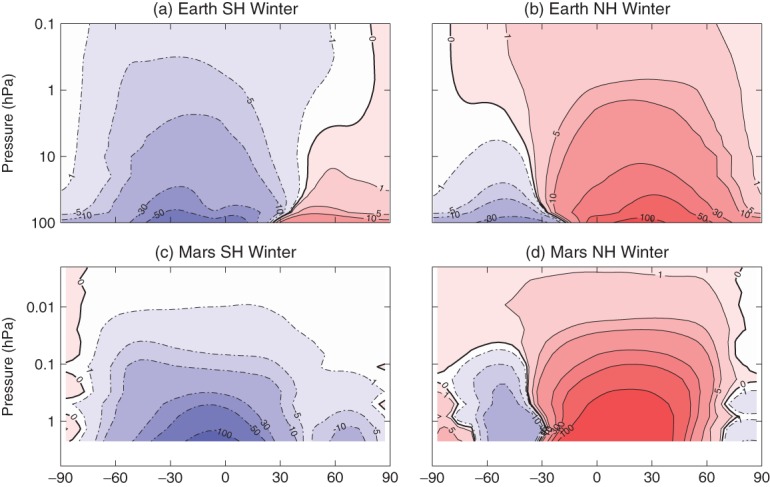
Seasonal mean TEM stream function calculated from 2 h data for (a, b) Earth and (c, d) Mars during (a, c) SH winter and (b, d) NH winter. Contours have units of kg m^−1^s^−1^ and are spaced logarithmically. See the text for winter definition. Dashed contours represent negative values.

In order to interpret the meridional circulation in terms of tracer transport, it is useful to consider the residual (Lagrangian) MSF, which on Earth is known as the predominantly wave-driven Brewer–Dobson circulation (Brewer, 1949). This is most appropriately defined using the TEM equations (Andrews *et al.*, 1987; Seviour *et al.*, 2012), such that


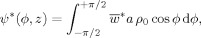
(9)

and


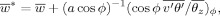
(10)

where 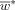 represents the residual vertical wind velocity. An overbar represents the zonal mean and a prime the deviation from it. We use 2 h data to compute *ψ* for the Martian reanalysis, thereby taking into consideration the diurnal tides, although results are similar if daily-mean data are used instead. *ψ*^∗^(*ϕ,z*) has been shown to provide a good approximation to the Lagrangian mean overturning circulation, at least outside the Earth's stratospheric surf zone (Pendlebury and Shepherd, 2003). Figure 3 shows this for Earth and Mars in the SH and NH. For Earth, the stream function in both hemispheres agrees well with a similar analysis using ERA-Interim reanalysis data performed by Seviour *et al.* (2012) which extended to 1 hPa, although here we are able to calculate *ψ*^∗^(*ϕ,z*) up to 0.1 hPa which shows the residual circulation continuing even beyond this level, which is into the lower mesosphere.

When compared with Mars, *ψ*^∗^(*ϕ,z*) is remarkably similar. This result suggests that large-scale tracer transport is similar on both planets at comparable levels in their respective atmospheres, with one large cell extending from the summer hemisphere all the way to the winter Pole, and, at least for the NH winter panel (d), a smaller counter-rotating cell solely in the summer hemisphere. Another interesting point is that for Mars, *ψ*^∗^(*ϕ,z*) is very similar to *ψ*(*ϕ,z*) (i.e. comparing Figure 2(e, f) with Figure 3(c, d)), suggesting that it is not necessary to use the TEM circulation when considering the overturning circulation on Mars. It also implies that upward propagating wave activity is not an important driver of the Martian meridional circulation, otherwise the TEM and Eulerian stream functions would differ, as on Earth.

### 3.2. The polar vortices

**Figure 4:**
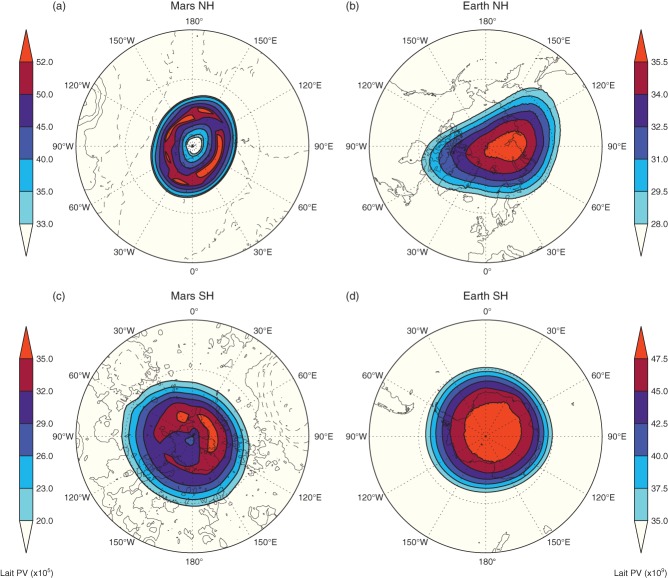
Polar stereographic maps of winter-averaged Lait PV for (a, c) Mars on the 350 K isentropic surface (∼ 40 km) and (b, d) Earth on the 850 K isentropic surface (∼ 30 km) for (a, b) the NH and (c, d) the SH. Only PV inside the vortex region is plotted (i.e. where 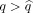). PV has been reversed in sign in the SH to be directly comparable with the NH. Note that colour scales are different for each panel. On Earth, the continent outlines are shown. On Mars, the elevation from the areoid (similar to the geoid on Earth) is plotted, with contours every 2 km elevation and negative values dashed.

Figure 4 shows winter averaged PV in the vortex region (over all the available years) for Mars on the 350 K isentropic surface and Earth on the 850 K isentropic surface for the NH and the SH. The winter period is defined as December–February and July–September for the Earth's NH and SH, respectively, and *L*_*s*_ = 200°–290° and *L*_*s*_ = 20°–110° for Mars' NH and SH, respectively. These are chosen such that the Earth's solstice coincides approximately with that on Mars. On Earth (Figure 4(b, d)), the asymmetry between the two vortices is clear and has been well documented (e.g. Waugh and Randel, 1999; Mitchell *et al.*, 2011) with the NH vortex being more elliptical, displaced further from the Pole and weaker than its SH counterpart. These differences arise due to the Earth's more variable orography and land–sea contrasts in the NH, which lead to more Rossby wave generation than in the SH (Andrews *et al.*, 1987), and are apparent throughout the depth of the stratosphere. On Mars, the vortices are also representative of all available isentropic surfaces (275–825 K), and most of the characteristics of the polar vortices are very similar, however, the vortex area does change dramatically with height. (This is examined in more detail later in the analysis.)

The most striking feature is that the NH polar vortex on Mars (Figure 4(a)) exhibits a distinct annular shape in PV, whereas on Earth PV is more often than not found to increase monotonically to the vortex centre (Figure 4(b, d)). Note that this is not always the case at higher altitudes (such as the mesosphere) where PV structures can be more complex (Harvey *et al.*, 2009). This annular nature is not so apparent in the Martian SH (Figure 4(c)), although a similar tendency is still present. The annular vortex could be thought of as a ring of high PV, surrounded by two opposing PV gradients. Hence the annular vortex satisfies the necessary condition for vortex barotropic instability, however this does not necessarily mean that the vortex will become unstable, especially considering the shallowness of the gradient on the poleward side of the annulus. As noted in Deem and Zabusky (1978), it is the gradients (or contours) between areas of uniform vorticity which are important for the distribution of PV in an annular vortex. Stability of an annular-type vortex can be sustained by external forcing, such as associated with the constant descent near the Poles of air in the meridional circulation, although it is non-trivial to demonstrate this mathematically.

Previous studies have been able to reproduce some form of stable annular vortices, for instance from theoretical models of shielded Rankine vortices (Dritschel, 1986; Harvey *et al.*, 2011), or in rotating annulus experiments (initialised with a differentially rotating annular disk; Barbosa Aguiar *et al.*, 2010), although these were actually barotropically unstable and their stationary ‘stable’ state was represented by a chain of smaller vortices (meso-vortices). However, no such studies have been specific to the distribution of PV observed in the Martian polar vortex case. Thus, for this analysis, we tested the stability of representative NH Martian vortices during different times in the Martian winter using a simple 2D Euler model, as in Harvey *et al.* (2011). However, after a few time steps, the vortices would always become unstable and ultimately break down, suggesting that such a model is not complex enough to adequately describe the Martian vortex system. While beyond the scope of this study, the explanation for this annular nature of the vortex as observed in the MACDA reanalysis requires more investigation, and may involve the representation of strong radiative forcing associated with the poleward extent of the descending branch of the Martian Hadley cell (Figure 2(e, f)). The NH vortex is also stronger than the SH vortex, which could be linked to its annular nature, but importantly this could lead to the vortex being a strong barrier against, e.g. dust transport to high latitudes, ultimately playing a role in intensifying latitudinal temperature gradients. The stronger PV gradients on the vortex edge may also help to facilitate Rossby wave propagation to greater altitudes. Finally, the NH vortex is more elliptical than one might expect. Throughout the winter the major axis of the vortex is aligned along the 30°W–150°E plane, implying a dynamical influence from a stationary wavenumber-2 wave aligned with the hemisphere-scale topography.

In the SH (Figure 4(c)), the Martian winter vortex is less distinct and weaker, and individual PV maps show more variability than in the NH (analysis not shown).

**Figure 5:**
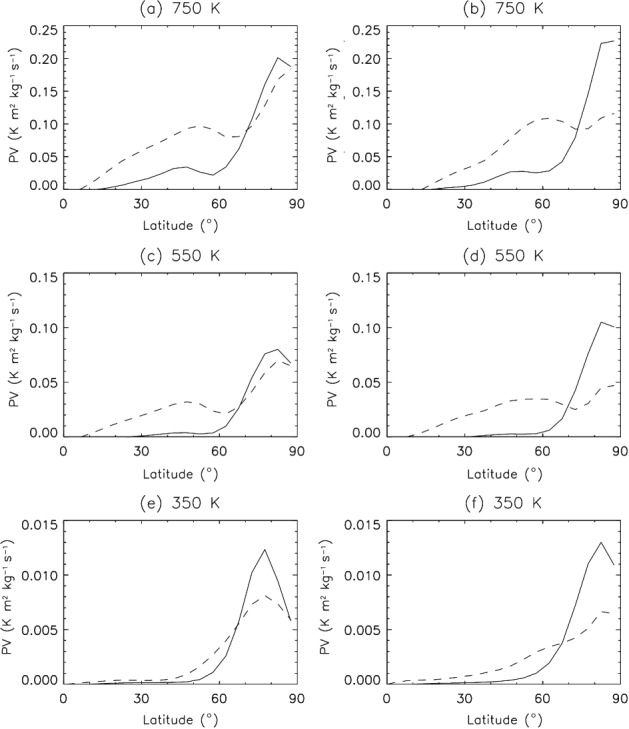
Winter-mean zonal-mean potential vorticity on the isentropic surfaces of (a, b) 750 K (∼ 70 km), (c, d) 550 K (∼ 60 km) and (e, f) 350 K (∼ 40 km) for (a, c, e) MACDA and (b, d, f) the Mars GCM forced by observed column dust opacities. Potential vorticity in the SH (dashed lines) has been reversed in sign to compare directly with the NH (solid lines).

To develop an overall picture of the annular nature of the polar vortices on Mars, we consider composites of the zonal-mean PV as a function of latitude for three isentropic surfaces which span the depth of the Martian atmosphere (Figure 5(a, c, e)). It is clear that the annular nature of both the NH and SH vortices is stronger at the lower levels, and this is particularly true for the NH. This is especially true as the zonal mean of the vortex masks, to some extent, the annular nature of the PV field. Figure 5(b, d, f) show the same analysis but for the freely running atmospheric simulation (without assimilating temperatures) forced by observed column dust opacities. The purpose here is to determine if the free-running GCM with the same dust distribution produces an annular vortex spontaneously. Figure 5 shows that the vortex is far less annular in nature in the simulations with no assimilated temperatures (b, d, f) than those with assimilated temperatures (i.e. the full MACDA product (a, c, e)). Therefore, there is something about the assimilated temperatures that enhances the annular nature of the vortex, which is not represented in the free-running model; this might imply additional radiative or dynamical perturbations to the Martian polar regions, which are excluded from our run with the MGCM with only assimilated dust, but implicitly included when assimilating retrieved TES temperature profiles. The effects of topography and orbital eccentricity cannot be considered responsible, since these effects are already taken into account in the Mars GCM, and in any event they do not seem to favour the annular shape of the vortex, as shown in Figure 5. We have also carried out a free-running GCM simulation where the orbit was forced to be circular (with radius equal to the aphelion distance) and the dust distribution was kept similar to the MACDA one, but no remarkable annular shape could be discerned (analysis not shown). Obviously, a caveat of such an experiment is that the dust distribution is not expected to be the same in the case of a circular orbit. With this experiment we can only observe that the change in direct absorption of short-wave radiation at perihelion (which on Mars occurs near the NH winter solstice) does not seem to produce a significant impact on the shape of the NH PV profile.

Other caveats for the dust distribution we have in both MACDA v1.0 and the GCM run with only assimilated dust is that retrievals of column dust opacity in the polar night (at high latitudes in winter) are generally not available and have to be prescribed. Furthermore, the vertical dust distribution is also artificially prescribed (Montabone *et al.*, 2014, gives details) and may not be realistic. While the assimilation of temperatures can alleviate errors in the prescribed dust, these might be present in runs without temperature assimilation and lead to errors in the radiative cooling of the atmosphere during the polar night. The verification of such a hypothesis would require further detailed analysis.

Nonetheless, preliminary results from the assimilation of temperature retrievals and estimated column dust opacities from the MCS radiometer (Kleinböhl *et al.*, 2009) on board NASA's Mars Reconnaissance Orbiter during MYs 28, 29 and 30 (2006–2011) show a peak at high latitudes in both winter hemispheres in the climatological PV profiles, more prominent around 350 K (not shown here). This is consistent with what is observed in the TES MACDA assimilation. MCS limb observations have higher vertical resolution and extension than TES nadir observations, allowing MCS thermal profiles to be retrieved up to 80 km altitude with twice the resolution of TES profiles. The fact that an annular shape for the Martian vortices is observed in the assimilation of two very different datasets at two different periods gives us some degree of confidence that this is a robust result. However, this may also mean that the reanalysis is more poorly constrained above 40 km; for that reason we primarily concentrate our analysis on levels below 40 km (except when analysing the full vertical structure of the vortex).

To develop an overall picture of how the vortices evolve through the winter period, we consider the moment diagnostics defined in Eqs (4) and (5). We compare time series of vortex moments of the Martian and terrestrial polar vortices on the 350 and 850 K isentropic surfaces, respectively. Note that differences in air density between Mars and Earth make it difficult to know which isentropic surfaces on Mars should be compared to those on Earth. We therefore take the approach that the levels chosen are both representative of a coherent polar vortex on each planet, and that it is the vortex variability that is interesting, rather than the absolute values.

**Figure 6:**
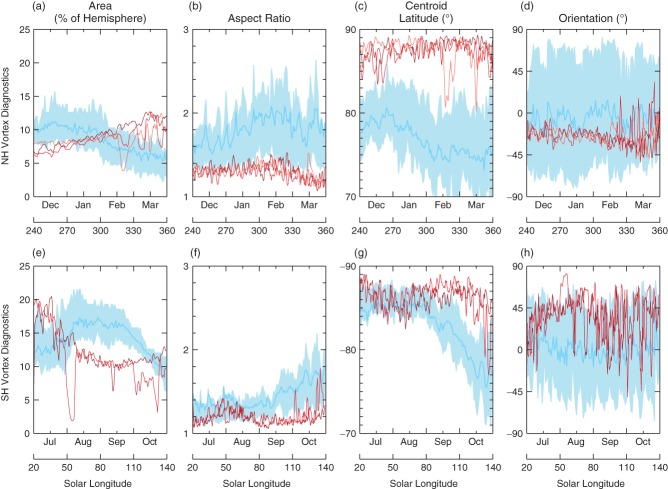
Winter time series of the vortex diagnostics for (top) the NH and (bottom) the SH. Red lines (Mars) show the MYs on the 350 K (∼ 40 km) isentropic surface, blue lines (Earth) show the mean and inter-quartile range of the terrestrial years on the 850 K (∼ 30 km) isentropic surface. The *x*-axis display both the terrestrial month (DJFM in the NH, and JASO in the SH), and the approximately equivalent Martian solar longitude (240–360°in the NH, and 20–140° in the SH).

Figure 6 shows time series of the moment diagnostics for Earth (blue) and Mars (red). The *x*-axis displays time during the winter season for both Mars (*L*_*s*_ = 240°–360° in the NH, and *L*_*s*_ = 20°–140°in the SH) and Earth (December to March in the NH, and July to October in the SH). For a meaningful comparison of the vortex area, it is expressed as a percentage of the surface area of the hemisphere. At these particular altitudes, the vortex area (Figure 6(a, e)) is approximately 10% of the hemispheric area for both Mars and Earth. The size and variability associated with these vortices may well be governed by the poleward extent of the meridional overturning circulation, the Equator-to-Pole temperature gradient and planetary wave bombardment of the vortex from the vertically propagating waves originating from the surface (and in the case of Earth, propagating through the troposphere). Many different climatic features can alter these influences (such as aerosol loading, temperature anomalies, orography) and each one can influence the others in complex, nonlinear ways.

On Earth, the asymmetry in tropospheric Rossby wave generation between the NH and SH is the primary cause of differences in the size of the vortices. Less wave activity in the SH allows for the vortex to tend towards its natural radiative equilibrium state, and not be weakened by wave–mean flow interactions, as is the case in the NH (Andrews *et al.*, 1987).

On Mars, there also exists an asymmetry in the vortex size between the two hemispheres. This is most likely associated with the asymmetric nature of the Martian Hadley circulation (Figure 2). In the NH winter, the descending branch of the Hadley circulation is further poleward than in the SH winter, hence the edge of the vortex is more poleward.

Of particular interest in the vortex area diagnostic is the occasional large shift observed in some of the MYs. Notably in the NH, large variability is observed at a solar longitude of *L*_*s*_ ∼ 320°(this particular year is MY 26; ∼2002–2004) and in the SH as *L*_*s*_ ∼ 50° and 110°. These large departures in vortex area are common features of terrestrial SSW events (Mitchell *et al.*, 2011) and may prove to be invaluable in understanding such phenomena in more detail. Of particular interest is the NH Martian event observed at *L*_*s*_ ∼ 320°, which shows both a large departure in the vortex area and an equatorward shift in the vortex centroid latitude (Figure 6(a–d)). This particular shift is of the order ∼8° equatorward, and Mitchell *et al.* (2011) show that this was indicative of a vortex displacement event on Earth§. The remaining diagnostics of Figure 6 show no SSW-like features. However, it is interesting to note that both the Martian NH and SH share many similar characteristics with the terrestrial SH vortex, particularly the vortex aspect ratio, which is consistently less than 1.5 for all three cases, and the vortex centroid latitude, which is consistently poleward of 85°. One notable difference between these three is the variability in the orientation of the Martian NH vortex (Figure 6(d), red lines). The variability of this particular diagnostic is greatly reduced compared to the Martian SH, or the terrestrial vortices. One possible explanation is that the vortex is dynamically locked to the underlying topography via large-amplitude stationary planetary waves, as seems likely when compared with Hollingsworth and Barnes (1996).

The exact mechanisms behind SSW events on Earth are still inadequately understood, although such events are observed to be strongly correlated with high levels of momentum deposition from vertically propagating planetary-scale waves (McIntyre and Palmer 1983). On Mars, it is still not clear to what extent the RPW mechanisms are dynamically or radiatively driven (Barnes and Hollingsworth 1987; Wilson 1997; Kuroda *et al.*, 2009). However, at *L*_*s*_ ∼320° in MY 26 a dust storm developed in the NH on Mars, causing localized heating. This storm belongs to the category of cross-equatorial storms (or ‘flushing storms’), which spread southward following the low-level Hadley cell flow in Equator-crossing western boundary currents (Joshi). In the SH, winds enhanced by temperature gradients associated with the dust front and topographic slopes, in addition to convective processes, made the storm rapidly attain a regional scale. The associated heating could have caused a subsequent alteration of the Hadley circulation (Wang, 2007). Some of the large deviations in the vortex characteristics for Mars are likely to be due to these changes in the Hadley circulation, which then lead to rapid polar warming events.

**Figure 7:**
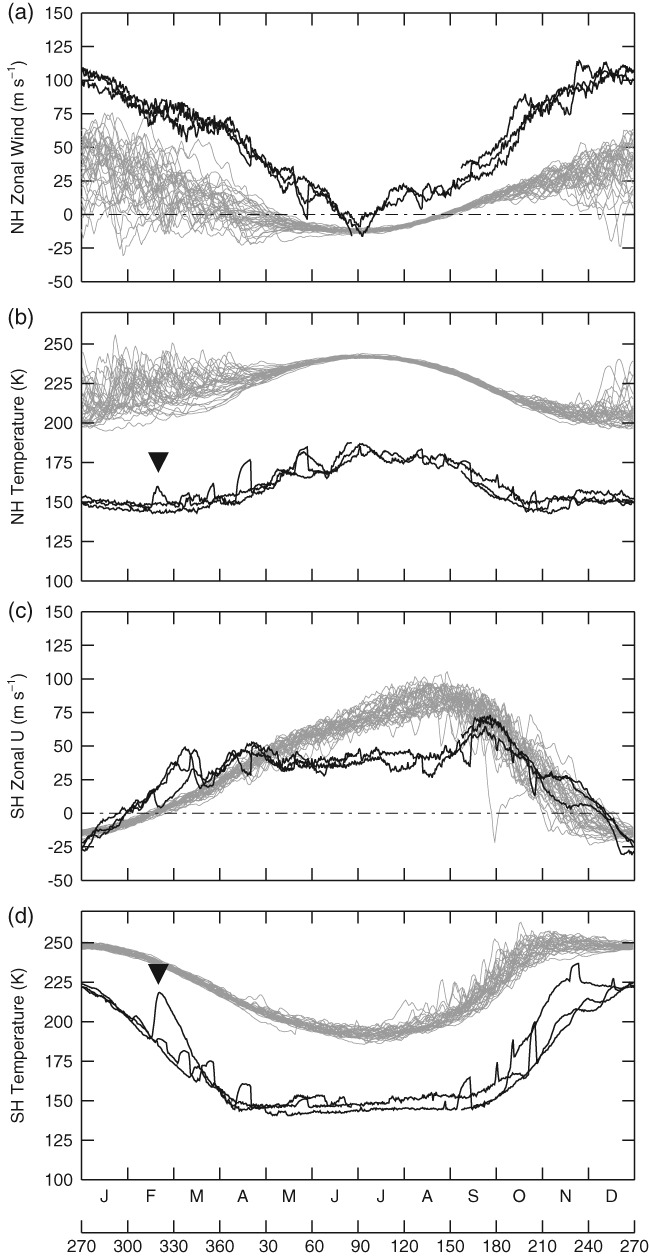
Time series of the zonal mean zonal wind speed at (a) 60°N and (c) 60°S and cap temperature averaged over (b) 60–90°N and (d) 60–90°S. Variables are all measured at the bottom of the polar jet; for Earth (grey lines) this is at 10 hPa, and for Mars (black lines) this is at 1 hPa. The dashed line marks zero wind. Solid triangles mark a specific dust storm on Mars (see text for details).

Polar warming events on Earth (SSWs) are often defined in terms of daily zonal mean zonal wind at 60°N (or 60°S in the SH) at 10 hPa, which is directly below the jet maximum. Figure 7(a, c; grey lines) show this diagnostic for all years (1979–2012) in the Earth reanalysis dataset, where (b) and (d) show also the polar cap temperature at the same height. The black lines show the same diagnostics but for Mars, and at 1 hPa (also the lower flank of the jet). On Earth the zonal winds and polar cap averaged temperatures are far more variable in winter than in summer, and this is because Rossby waves cannot propagate in the summer easterly wind regime (Charney and Drazin, 1961). The Martian zonal winds and cap temperatures (black lines) seem to be less variable than the corresponding terrestrial diagnostics during winter, although we note that it is hard to draw any strong conclusions as there are only three years of data available. The largest deviations on Mars are observed in the SH cap temperature (d), and are each associated with dust storm events (Wang, 2007). The dust storm at *L*_*s*_ ∼ 320° is marked with a solid triangle, and can be clearly seen as a cap temperature perturbation in both the NH and SH. In particular the NH polar cap temperature rises by ∼10 K, comparable with minor SSW events on Earth.

**Figure 8:**
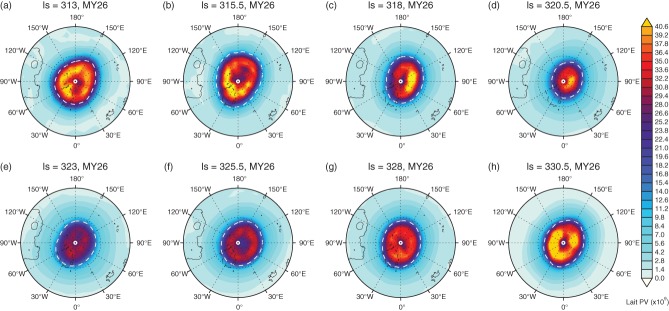
Time evolution of the NH Lait-transformed PV on Mars for the 350 K (∼ 40 km) isentropic surface during the dust storm of MY 26. Contours show the elevation from the aeroid. The vortex edge is superimposed as a dashed white line.

One issue when dealing with zonal mean and cap-averaged diagnostics is that the vortex is inherently complex; these diagnostics cannot possibly capture all of the key features of the vortex structure. Therefore one must study the vortex in the full 2D sense (i.e. varying in longitude and latitude) to fully understand the vortex evolution under, e.g. dust storm conditions. Figure 8 shows a time evolution of Lait-transformed PV on the 350 K surface during the MY26 dust storm, which lasted for only about 10°*L*_*s*_ (∼ 5 Earth days), most likely due to the season departing from winter solstice (Wang, 2007). The annular nature of the PV is clearly observed up until *L*_*s*_ = 318° where the PV is weakened and shifted equatorward, much like a terrestrial SSW event. The vortex continues to weaken, reaching a minimum at *L*_*s*_ = 323°, from which point it recovers. The recovery of the polar vortex on Mars is much faster than on Earth, presumably due to the faster radiative time-scales. One clear difference between daily averaged PV maps on the two planets is the lack of PV filamentation on Mars. This may be an artifact of the resolution of the MACDA data, although similar resolution data of the terrestrial polar vortices still exhibit filamentation. On Earth, PV filaments are constantly stripped away from the main vortex air mass and mixed into the background flow, and this is due to the interaction of the vortex with large-scale Rossby waves, a process that seems to be absent in the MACDA reanalysis dataset.

**Figure 9:**
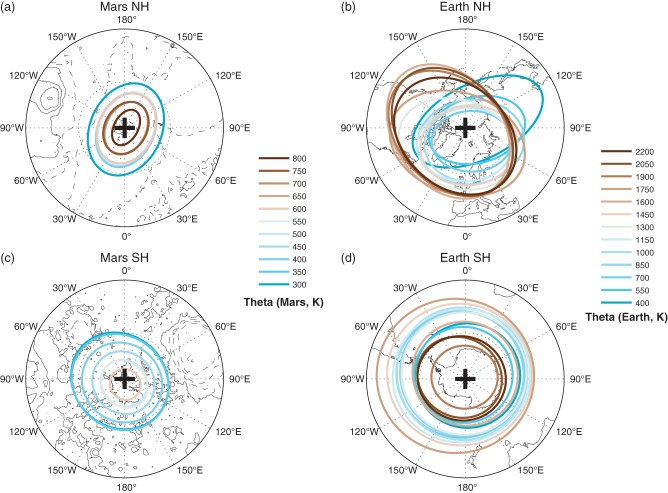
Average vortex equivalent ellipses during a typical winter month on a range of theta surfaces in the (a, b) NH and (c, d) SH for (a, c) Mars and (b, d) Earth. The isentropic surface is indicated by the changing colours and displayed in the key below the relevant panels. The typical winter months are: Earth NH, December; Earth SH, July; Mars NH, *L*_*s*_ = 270°–320°; Mars SH, *L*_*s*_ = 90°–140°. On Earth, the continent outlines are shown. On Mars, the elevation from the areoid is plotted, with contours every 2 km elevation, with negative values dashed.

It is important to note that, while we have mainly considered the polar vortex at a single vertical level, it is actually a vertically coherent structure. This is especially important as SSW events are known to have a complex vertical evolution (Matthewman *et al.*, 2009). Figure 9 shows the climatological structure of the polar vortices on Mars and Earth for the NH and the SH. The ellipses represent the vortices on the displayed isentropic level, which are determined using the diagnostics detailed in section 2.2, and previously displayed for one surface in Figure 6.

A clear difference between the two planets is that on Mars the vortices become dramatically smaller with height, to the extent that in the NH the top of the vortex is located around *θ* = 800 K (∼ 70 km) and in the SH the top is around *θ* = 650 K (∼ 60 km). This can also be observed in the winter-averaged zonal mean wind analysis (Figure 2), which shows the poleward tilt of the jet with height. In contrast, on Earth (Figure 9(b, d)) the area of the vortices increases with height, although in the SH the vortex maximum area appears in the upper stratosphere, at which point the vortex becomes more conical like the Martian vortex. (Note that this result hold for all months in which the SH polar vortex is well established on Earth.) As such, the vertical structure of the terrestrial vortex is far more complex, but is still well established throughout the depth of the stratosphere. For instance, there is a notable rotation of the terrestrial vortex with height in the NH (Figure 9(b)) which is completely absent from the Martian polar vortices. The Martian polar vortices remain remarkably coherent with height, even on day-to-day time-scales (not shown) where the orientation of the vortex can vary dramatically during certain times of the season (e.g. Figure 6 (right)). While the zonal phase does not change with height for both the Martian NH and SH, this effect is most apparent in the NH such that the vortex shows the same elliptical form and orientation as the vortex on a single surface (350 K) observed throughout the NH winter (Figure 4). In the NH there is a clear wave-2 pattern in the vortex structure, which extends throughout the depth of the atmosphere and is consistent with the surface pressure patterns (Hollingsworth and Barnes 1996). Similarly, in the SH, the vortex has a wave-1 structure, which is consistent with stationary planetary wave forcing generated from the surface topography (particularly the Hellas crater).

In the Martian SH, the vortex remains more circular than in the NH. On Earth the ellipticity of the NH vortex gradually decreases with height, consistent with Mitchell *et al.* (2011). It should be noted that in the SH on day-to-day time-scales the aspect ratio is ∼ 1.2 (Figure 6) but, due to smearing caused by averaging over multiple days, the July monthly mean vortex (Figure 9(d)) has an aspect ratio of ∼ 1 at most heights.

## 4. Concluding remarks

In this study we compared and contrasted the structure and evolution of the polar vortices on Mars with those on Earth. Using one of the first Martian reanalysis datasets, we have analyzed the mean state and variability of the Martian polar vortices to determine how similar in nature they are to the terrestrial polar vortices. In doing so, we have provided the first step to determine if useful analogies between SSWs on Earth and rapid polar warmings on Mars can be drawn. Specific dust storm events on Mars and their impact on the polar vortex structure will be addressed more comprehensively in future work.

The key differences and similarities between the terrestrial and Martian polar vortices are:

1. The residual meridional winter circulation is found to be very similar on both planets, and in both hemispheres. This also resembles the Eulerian circulation on Mars, but is sufficiently different from the Eulerian circulation on Earth.
2. While the polar vortices on both planets are characterised by strong circumpolar jets, within which are weaker winds, the PV structure is somewhat different. The PV field of the Martian polar vortices (and in particular the NH vortex) are shown to be annular in nature in the MACDA v1.0 reanalysis, whereas this is generally not the case for Earth. There is evidence for the stability of such vortices, but the causes of the annular nature are probably not due to the topography of Mars, or the ellipticity of the Martian orbit. The annular structure is primarily observed only when temperature retrievals are assimilated in the Mars GCM.
3. There is an asymmetry in the strength of the Martian polar vortices; the NH vortex is stronger than the SH vortex. A somewhat similar (but of opposite sense) asymmetry is also observed on Earth, although this is thought to be due to the interaction of more upward propagating Rossby waves in the NH than the SH.
4. The Martian polar vortices are consistently centred over the Pole at all heights, implying that the wave–mean flow interactions which ubiquitously displace the polar vortices equatorward on Earth are either not present on Mars, or are at least less dominant.
5. The variability in the structure of the Martian polar vortices is reduced during winter compared with the terrestrial polar vortices, and most variability seems to be related to radiative changes, linked to changes in dust loading, rather than dynamical changes, as observed on Earth.
6. The area of the polar vortices on Mars decreases dramatically with height, such that the Martian reanalysis captures the entire vertical extent of the polar vortex. This decrease in height is not, in general, observed on Earth, except towards the top of the polar vortex in the SH. Furthermore, a unique rotation of the NH terrestrial vortex is observed with height.
7. The one specific rapid polar warming event on Mars considered in this analysis corresponded to a dust storm observed during MY 26 around *L*_*s*_ ∼ 320° and showed similarities to terrestrial vortex displacement SSW events. In particular, an equatorward shift in the vortex air mass of ∼ 10° was observed, as well as an overall weakening of the vortex circulation. However, the specific mechanisms driving the extreme vortex variability are probably different between the two planets.

The analysis presented here has shown that, while both planets exhibit polar vortices, the structure (both horizontal and vertical) and evolution of their polar vortices are somewhat different. However, Rossby waves are generally stronger each side of the Martian winter solstice period than at other times in the year (Wang, 2007) and our analysis has shown that there do seem to be some interesting features which are analogous to terrestrial SSW events during this period (most likely related to dust storms). The most notable feature for interplanet comparison is the presence of an equatorward shift in PV during extreme vortex events on Mars, which on Earth is thought to play a key role in stratosphere–troposphere coupling (Ambaum and Hoskins, 2002; Mitchell *et al.*, 2013). We therefore conclude that, while there are large structural differences between the vortices on Earth and Mars, interesting insights are found concerning vortex variability, particularly under conditions of extreme radiative forcing from changes in the atmospheric aerosol composition.

### 4.1. Future directions

Two important questions regarding Martian polar vortex dynamics which still remain unresolved are:

- Why are the vortices in the MACDA reanalysis annular in nature?
- If they are a robust feature of the Martian atmosphere, why are such annular vortices not unstable?

To address the former, an extensive study of the Martian polar regions and their connections to the Tropics through the meridional circulation is required. The latter requires a more in-depth stability analysis of the evolution of the polar vortices than we were able to perform here.

Two limitations for Mars are the short period of available MACDA v1.0 reanalyses (only about three MYs) as well as its relative coarse spatial resolution (5° longitude × 5° latitude for 25 vertical levels extending from the ground to about 80 km, if one excludes the sponge layers).

Future work will try to reduce these limitations, by extending the reanalysis of the Martian atmosphere to the period covered by the Mars Climate Sounder on board NASA's Mars Reconnaissance Orbiter, and increasing the spatial resolution both in the horizontal and in the vertical. Further work is also required to establish how the radiative effects of the Martian dust compare with any analogous terrestrial aerosols (perhaps volcanic dust, nuclear fallout, or aerosols released during a geo-engineering project; e.g. Turco *et al.*, 1983). If these types of terrestrial aerosol are found to have similar radiative and dynamical effects, then Mars could prove to be a useful natural laboratory for studying the possible impacts of any future aerosol-based geo-engineering proposal.

## Footnotes

* We use the term ‘rapid polar warming’ to refer to an event on Mars which may be SSW-like

† Clancy *et al.*, (2000) proposed the convention to numerate the Martian years beginning at the Martian northern spring equinox preceding the observations of the planet-encircling dust storm in 1956. Therefore MY 1 starts on 11 April 1955.

‡ We use a Lambert projection rather than the polar stereographic projection used by Waugh and Randel (1999) as it is area-conserving.

§ A vortex displacement event is a type of SSW where the vortex air mass is shifted equatorward before often completely breaking down and mixing out into the background flow.

## Appendix

**Table A1:** Terms and acronyms used in this analysis

Term	Meaning
Areoid	An imaginary sphere with a constant radius of 3396 km from the centre of Mars.
Displacement	A type of SSW event dominated by wavenumber-1 Rossby waves.
ECMWF	European Centre for Medium-Range Forecasting.
GCM	Global Climate Model.
MACDA	Mars Analysis Correction Data Assimilation; reanalysis for Mars.
MERRA	Modern Era Retrospective ReAnalysis; reanalysis for Earth.
MCS	Mars Climate Sounder.
MGCM	Mars Global Climate Model.
MGS	Mars Global Surveyor.
MSF	Mass stream function.
MY (24, 25, 26)	Martian Year.
NASA	National Aeronautics and Space Administration.
NH	Northern Hemisphere.
Polar-night jet	The stratospheric jet on Earth which marks the approximate polar vortex edge.
Pseudo-altitude	The height from the surface taking into account the surface topography.
PV (Lait)	Potential vorticity, scaled by the Lait transform to account for changes in PV at different heights.
RPW	Rapid Polar Warming.
SH	Southern Hemisphere.
Sol	A Martian day, of which there are 668.98 in a Martian year.
Solar longitude (*L*_*s*_)	The angle between Mars and the Sun, used to measure the time through the Martian year so that *L*_*s*_ = 90° corresponds to the NH summer solstice, *L*_*s*_ = 180° to the autumn equinox and *L*_*s*_ = 270° to the winter solstice.
Split	A type of SSW event dominated by wavenumber-2 Rossby waves.
SSW	Sudden Stratospheric Warming.
TEM	Transformed Eulerian mean.
TES	Thermal Emission Spectrometer.
